# Effects of Pecking Materials Provision and Edibility on Productivity Indicators in Laying Hens: A Systematic Review and Meta-Analysis

**DOI:** 10.3390/ani16193062

**Published:** 2026-09-29

**Authors:** Woo-Do Lee, Yejin Mun, Youngjun Na, Han Tae Bang, Chan Ho Kim

**Affiliations:** 1 Animal Welfare Division, National Institute of Animal Science, Rural Development Administration, Wanju 55365, Republic of Korea; woodo92@korea.kr (W.-D.L.); myj0804@korea.kr (Y.M.); banght80@korea.kr (H.T.B.); 2 Department of Animal Science, Jeonbuk National University, Jeonju 54896, Republic of Korea; 3 Department of Animal Science and Technology, Konkuk University, Seoul 05029, Republic of Korea; ruminoreticulum@gmail.com

**Keywords:** egg productivity, environmental enrichment, laying hens, meta-analysis, pecking material

## Abstract

Feather pecking and cannibalism arise when laying hens’ foraging needs are not satisfied, reducing productivity, increasing stress, and elevating mortality. Providing pecking materials is a welfare measure that redirects pecking from conspecifics to the provided material, and this meta-analysis evaluated its effects on productivity and mortality in laying hens. Following the PRISMA guidelines, a systematic search of PubMed, Google Scholar, and ScienceDirect identified 18 articles comprising 24 experiments that examined the effects of pecking materials on body weight (BW), egg production rate (EPR), egg mass (EM), average daily feed intake (ADFI), egg weight (EW), feed conversion ratio (FCR), and mortality (MORT). Risk of bias, assessed using the SYRCLE tool, was generally low to unclear across most domains, though blinding of caregivers was rated as high risk in all studies. A random-effects model was used to pool standardized mean differences (SMD) across studies, and subgroup analysis based on material edibility was conducted through meta-ANOVA to explore potential sources of heterogeneity. Overall, pecking materials provision decreased both BW (SMD = −0.59; 95% CI: [−1.03, −0.14]) and MORT (SMD = −1.10; 95% CI: [−1.79, −0.42]). Subgroup analysis revealed contrasting BW patterns by edibility (edible: decreased; non-edible: increased), whereas MORT decreased consistently regardless of edibility. Publication bias was detected for EPR and ADFI, but trim-and-fill adjustment did not alter the overall conclusions. These findings indicate that pecking materials provision is a useful strategy that reduces mortality without compromising productivity, and suggest that material edibility may partly explain the inconsistent results reported in previous studies. However, given the substantial heterogeneity and unclear risk of bias observed in several domains, further studies incorporating a broader range of material types and rearing conditions are needed to clarify the sources of variability.

## Simple Summary

Feather pecking is a major welfare concern in the laying hen industry, causing stress and adversely affecting productivity and mortality. As a primary countermeasure against feather pecking, pecking materials have been provided to laying hens, but their effects have been reported inconsistently across studies. According to the present meta-analysis, the provision of pecking materials significantly reduced mortality, while productivity indicators were not significantly affected. In contrast, body weight responded oppositely depending on the edibility of the material, with edible materials decreasing and non-edible materials increasing body weight. Notably, the reduction in mortality was consistent regardless of material edibility. These findings suggest that pecking materials could serve as a useful welfare-enhancement strategy that reduces mortality while maintaining productivity in laying hens.

## 1. Introduction

Laying hens naturally exhibit complex social group behaviors and spend a substantial portion of their daily time budget engaged in ground scratching and exploratory foraging [1]. In modern egg production, however, intensive commercial housing systems impose spatial and environmental constraints that severely restrict the expression of these highly motivated, species-typical behaviors [2]. When such behavioral opportunities are thwarted, the lack of environmental complexity and adequate foraging substrates acts as a chronic welfare stressor [3]. Consequently, rearing hens in inadequate environments can severely compromise flock welfare and ultimately result in reduced overall productivity [4]. In particular, poor environments and limited rearing space fail to satisfy the hens’ innate foraging needs, redirecting this unsatisfied motivation into aberrant beak-related behaviors, most notably feather pecking (FP) [4,5]. While the modern poultry industry strives to maintain high productivity alongside optimal production efficiency, achieving this balance fundamentally requires addressing these environmental constraints and providing an appropriate level of behavioral welfare [6,7]. FP is a common problem in laying hens and is influenced by environmental factors [8,9]. Importantly, cannibalism is not an inevitable or ubiquitous trait across all laying hen flocks; rather, it manifests as an aberrant behavioral pathology triggered by specific environmental, behavioral, and nutritional stressors, such as mineral and trace element deficiencies (e.g., sodium and dietary fiber), excessive stocking density, inadequate foraging substrates, and excessively high light intensity [10]. In the past, damage caused by FP and cannibalism was controlled by beak trimming, but this practice has been regulated or banned in many countries for welfare reasons [9]. As an alternative, providing pecking materials, one of the environmental enrichment (EE) methods, can help stimulate natural behaviors in poultry while relieving negative emotions such as fear and depression and reducing FP behavior [11]. Providing various EE, including litter, will help to direct foraging and exploration behaviors towards harmless substrates, thereby reducing FP, severe feather pecking (SFP), and vent pecking (VP) in laying hens [12]. In line with these mechanisms, numerous studies have examined the effects of various pecking materials on the welfare of laying hens. Alfalfa bales and pumice stones did not significantly affect feather condition in a commercial rearing environment, but were associated with reduced serum corticosterone, lactate dehydrogenase, and creatinine, indicating improved physiological stress [11]. Weekly provision of novel objects such as balls, pet toys, and plastic pipes, by contrast, increased exploratory behaviour and redirected pecking toward these objects, improving feather and beak condition and reducing comb wounds, thereby mitigating physical damage [13]. The provision of live black soldier fly larvae similarly reduced feather pecking and consequently improved plumage condition, as the live insects served as a natural substrate for pecking [14]. While these findings collectively support a beneficial effect of pecking materials on welfare, their effects on productivity remain inconsistent across studies. Alfalfa bales and pumice stones were reported to reduce floor-laid eggs and improve productivity and egg quality [11], whereas another study using the same materials found no effect on egg production rate and instead an increase in floor-laid eggs [15]. Similarly, mixed provision of edible pecking materials such as mealworms, cabbage, corn, and rose hips produced effects that varied depending on whether the birds had previously experienced early-life stress [16]. Recently, various EEs such as hay (alfalfa bale), pumice stones, balls, toys, and insects (mealworms, black soldier flies, etc.) have been investigated to provide appropriate animal welfare levels while improving productivity, but it is showing inconsistent results [11,13,14,15,16].

Quantitative, data-integrative approaches have also been increasingly applied across diverse areas of biological and biomedical research to derive robust conclusions from complex datasets [17,18]. Among such approaches, meta-analysis is a factual technique and robust statistical method that utilizes data from past studies to derive appropriate and useful conclusions based on research questions [19,20]. In summary, the characteristics and advantages of meta-analysis are as follows [21,22]: (1) It is a quantitative method with more powerful analysis power by integrating multiple independent studies into statistical analysis; (2) it can measure sample size and potential effects by combining multiple research results; (3) it can prevent subjective interpretation because it summarizes the results using an objective formula; (4) it can derive systematic responses according to the diversity of publications by integrating various variables; (5) unlike a single experiment that only reflects experimental conditions, meta-analysis systematically considers heterogeneity between studies; (6) it can convert research results into applicable knowledge; and (7) it is a very useful method for identifying and verifying future research topics. These characteristics necessitate the use of meta-analysis techniques to resolve conflicting findings among studies addressing the same objectives and to reach valid conclusions with stronger analytical power [23]. Although previous meta-analyses have examined the effects of environmental enrichment on feather pecking and feather damage in laying hens [24], to our knowledge, none has quantitatively synthesized its effects on productivity and mortality.

Therefore, the purpose of this study was to quantify the effect of pecking materials provision, compared with no provision, on productivity indicators in laying hens. The productivity indices of laying hens that we analyzed were body weight (BW), egg production rate (EPR), egg mass (EM), average daily feed intake (ADFI), egg weight (EW), feed conversion ratio (FCR), and MORT. Our research results are expected to provide systematic information on EE provision to laying hens and serve as a database to maximize egg productivity and welfare levels.

## 2. Materials and Methods

### 2.1. Literature Search Strategy

The literature search for this study was conducted using electronic databases such as PubMed, Google Scholar, and ScienceDirect from 30 September 2024 to 17 February 2025, with the search restricted to literature published up to 2024. The search terms included a combination of keywords: ‘laying hens’ or ‘layers’, and ‘EE’ or ‘pecking materials’ or ‘pecking objects’ or ‘novel object’, and ‘productivity’ or ‘egg production’. These terms were selected to capture studies examining the provision of pecking-related enrichment materials and their effects on productivity outcomes in laying hens. The literature search was conducted independently by two researchers. The articles were selected based on the title and abstract content, and then the selected records were compared to reach a consensus on the eligibility criteria. The selected articles were read in their entirety, and quantitative laying hen performance data were extracted to perform a meta-analysis. If no agreement was reached, another researcher would conduct an in-depth analysis to resolve whether or not to use it.

### 2.2. Selection Criteria

This systematic review followed the Preferred Reporting Items for Systematic Reviews and Meta-Analyses (PRISMA) 2020 guidelines [25]. The literature used in our meta-analysis met the following criteria: (1) A study comparing a group that was provided with EE (treatment) and a group that was not provided (control); (2) case studies analyzing indicators of laying hen productivity by providing pecking materials as an EE; (3) at least one of the productivity indicators (BW, EPR, EM, ADFI, EW, FCR, and MORT) according to the provision of pecking materials must be provided; and (4) studies that provided the information necessary to conduct the analysis (experimental design, mean, standard deviation (SD), standard error of the mean (SEM), number of replicates).

**Figure 1:**
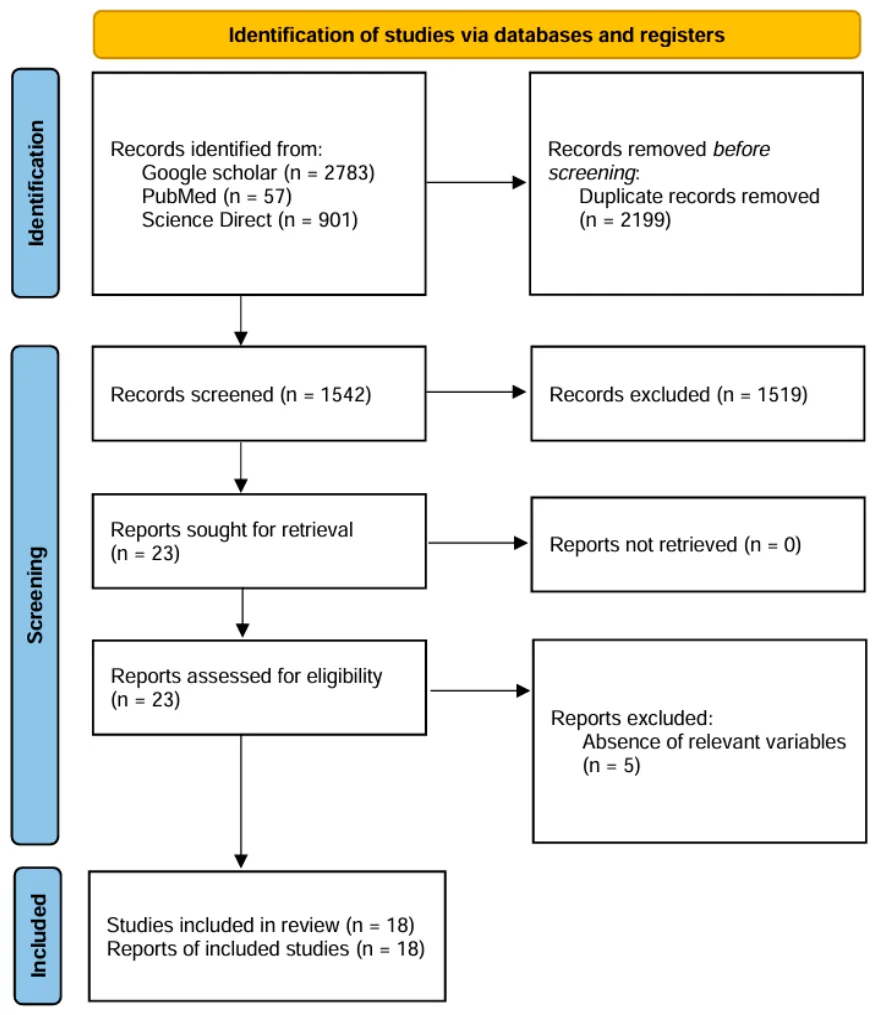
PRISMA flow diagram of the process of literature selection. Consider, if feasible to do so, reporting the number of records identified from each database or register searched (rather than the total number across all databases/registers).

On the other hand, the exclusion criteria are as follows: (1) Non-original studies, such as reviews, letters, and commentaries; (2) cases investigating the effects of EE through methods other than providing pecking materials; (3) studies that simultaneously provided other types of objects, such as perches or claw abrasives, in addition to the pecking materials; (4) a study that did not analyze any of the productivity indicators of laying hens; (5) literature that did not provide information on repetitions, SEM, or SD; and (6) studies written in a language other than English, where the reviewers determined that accurate interpretation of the content or extraction of data could not be reliably performed. Results by indicator (positive or negative effect) were not considered as selection criteria for including papers in the database. Based on these criteria, the final 18 selected articles, comprising data extracted from 24 experiments, were used for the analysis (Figure 1).

### 2.3. Assessment of Risk of Bias

Risk of bias was assessed using Systematic Review Centre for Laboratory Animal Experimentation (SYRCLE)’s Risk of Bias (RoB) tool for animal studies [26], which was developed based on the Cochrane RoB tool and adapted to incorporate items applicable to animal intervention studies. This assessment was independently conducted by two reviewers, and discrepancies were resolved through discussion or consultation with a third reviewer. For each of the eligible studies included in this review, a total of ten domains corresponding to six types of bias were evaluated: selection, performance, detection, attrition, reporting, and other bias. Each domain was judged as “Low risk,” “High risk,” or “Unclear.” Upon completion of all assessments, the overall proportion of risk of bias judgments across the included studies was presented as a bar plot.

### 2.4. Data Extraction

After the literature selection process, data were independently extracted by two reviewers using a standardized data extraction form, and discrepancies were resolved through discussion or consultation with a third reviewer. The extracted data included publication year, first author name, type of housing, species and age of laying hens for the experiment, beak trimming, timing of pecking materials provision, type of pecking materials provided and their edibility, and productivity analysis indicators (Table 1). Data from each study required to conduct meta-analysis were collected using appropriate procedures for collecting data. When a study reported outcome measurements at multiple time points, all reported values were extracted and included as separate experiments. However, comparisons in which pecking material provision was discontinued or introduced only during part of the observation period were excluded; only comparisons maintained throughout the entire observation period were included. The mean and standard deviation and sample size (n) between the control and experimental groups were extracted, and if the number of SD was not reported in the studies, the SD value was confirmed using the formula SD = SE × sqrt(n) [27]. All observation data were converted to the same units of measurement and analyzed, and in cases where data were presented graphically, the online extraction tool WebPlotDigitizer (version 4.6; Automeris LLC, Pacifica, CA, USA) was used to extract accurate data values [19].

### 2.5. Statistical Analysis

All statistical analysis were conducted in the R software environment (version 4.1.2; R Foundation for Statistical Computing, Vienna, Austria) using Rstudio (version 2021.091+372; Posit Siftware, PBC [formerly RStudio, Inc.], Boston, MA, USA) and performed using the “meta” package (version 5.2-0).

#### 2.5.1. Meta-Analysis

For each productivity indicator, only experiments reporting the necessary quantitative data (mean, SD, and sample size) for that outcome were included in the corresponding meta-analysis; therefore, the number of experiments included differed across indicators. To compare and pool results across studies employing different units and measurement scales, the standardized mean difference (SMD) between the control and treatment (EE) groups was calculated for each productivity indicator. Hedges’ g [28] was applied during SMD calculation to correct for bias associated with small sample sizes. Given the heterogeneity expected among the included studies, a random-effects model (REM) was used to pool the effect sizes, as this model accounts for both between-study variability (true heterogeneity) and within-study sampling error [29,30]. Pooled SMDs were reported with 95% confidence intervals (CI). SMD values of 0.2, 0.5, and 0.8 were considered to indicate small, medium, and large effects, respectively. In the forest plot, the dotted line within the diamond represents the pooled estimate; the overall pooled estimate was considered statistically significant when the corresponding 95% CI did not include zero. Individual effect sizes are represented by squares in the forest plot.

#### 2.5.2. Heterogeneity

Between-study heterogeneity for each variable was assessed using the tau squared (τ^2^; the amount of heterogeneity), Cochrane’s Q statistic *p*-value and Higgin’s *I*^2^ statistic (the percentage of total variation between studies due to heterogeneity). The τ^2^ represents the variance between studies and is a very common measure that can measure the heterogeneity between studies [31,32]. A larger τ^2^ indicates greater variability in the true effect sizes across the included studies and is also incorporated into the weighting of each study in the random-effects model [33]. For Q statistics, the power of the test was low because the number of studies providing the splitting materials was small, so heterogeneity was assessed at the level of *p*-value < 0.1 [34]. Higgin’s *I*^2^ was classified as low heterogeneity (*I*^2^ < 25%), moderate heterogeneity (25% ≤ *I*^2^ < 50%), high heterogeneity (50% ≤ *I*^2^ < 75%), and very high heterogeneity (*I*^2^ ≥ 75%) [35]. At this time, when the *I*^2^ value exceeded 50%, it was considered significant heterogeneity [36].

#### 2.5.3. Meta-ANOVA (Subgroup Analysis)

To identify potential sources of heterogeneity observed in the meta-analysis, meta-ANOVA was conducted based on the edibility of the materials of enrichment (edible vs. non-edible). Materials clearly intended for ingestion and with potential nutritional effects on laying hens, such as silage, alfalfa bales, crops and fresh produce (e.g., carrots, lettuce), were classified as edible. Mussel shells were also classified as edible, as the authors provided them for the purpose of supplementary calcium intake and reported the actual quantity ingested. In contrast, materials such as toys and pecking stones were classified as non-edible; although pecking stones may allow for some incidental mineral intake, their primary purpose is to satisfy pecking behavior rather than to provide nutrition. In studies where pecking stones and alfalfa were provided simultaneously, the treatment was classified as edible, given the potential nutritional contribution of the co-provided edible component.

#### 2.5.4. Publication Bias

Publication bias was assessed to evaluate the validity of the pooled results. For each productivity indicator, funnel plots were visually inspected, and Egger’s linear regression test was performed [37]. Funnel plot asymmetry combined with a significant result from the regression test was considered indicative of potential publication bias. For productivity indicators in which publication bias was identified, the trim-and-fill method was applied to estimate an adjusted pooled effect size [38].

## 3. Results

### 3.1. Overview of Included Studies

The study selection process, including the number of records identified, screened, and excluded at each stage, is presented in Figure 1. A total of 3741 records were identified from databases. Following screening, duplicate records, ineligible reports, and reports lacking data necessary for analysis were excluded, resulting in 18 studies being included in the meta-analysis. Because some studies included multiple enrichment materials as independent treatment groups or differentiated treatments according to the timing of provision, the 18 studies were divided into 24 experiments.

The characteristics of the 18 studies are summarized in Table 1. A variety of laying hen strains were used across the studies, with Hy-Line Brown (n = 7) and White Leghorn (n = 4) being the most frequently investigated. Excluding four studies that did not report beak-trimming status, the majority of studies were conducted on non-beak-trimmed birds (n = 8). Housing systems varied considerably across studies, including cage, pen, free-range, and multi-tier aviary systems. A range of environmental enrichment materials were provided to stimulate the hens’ pecking motivation, including edible substrates offering additional nutritional intake, such as silage and various crops, and non-edible objects intended to satisfy pecking behavior, such as key rings and string devices. In addition, several studies provided materials such as mussel shells or pecking stones, which are not directly ingested as feed but may provide supplementary mineral intake through abrasion during pecking. Most studies provided enrichment during the laying period (n = 12), whereas only four studies provided enrichment during the rearing period. Specifically, Schreiter et al. [15] reported the effects of enrichment provided continuously from the rearing through the laying period, while Schreiter et al. [39] employed a 2 × 2 factorial design examining the effects of enrichment timing, reporting outcomes for provision during rearing alone, laying alone, and across the entire study period.

### 3.2. Risk of Bias

**Figure 2:**
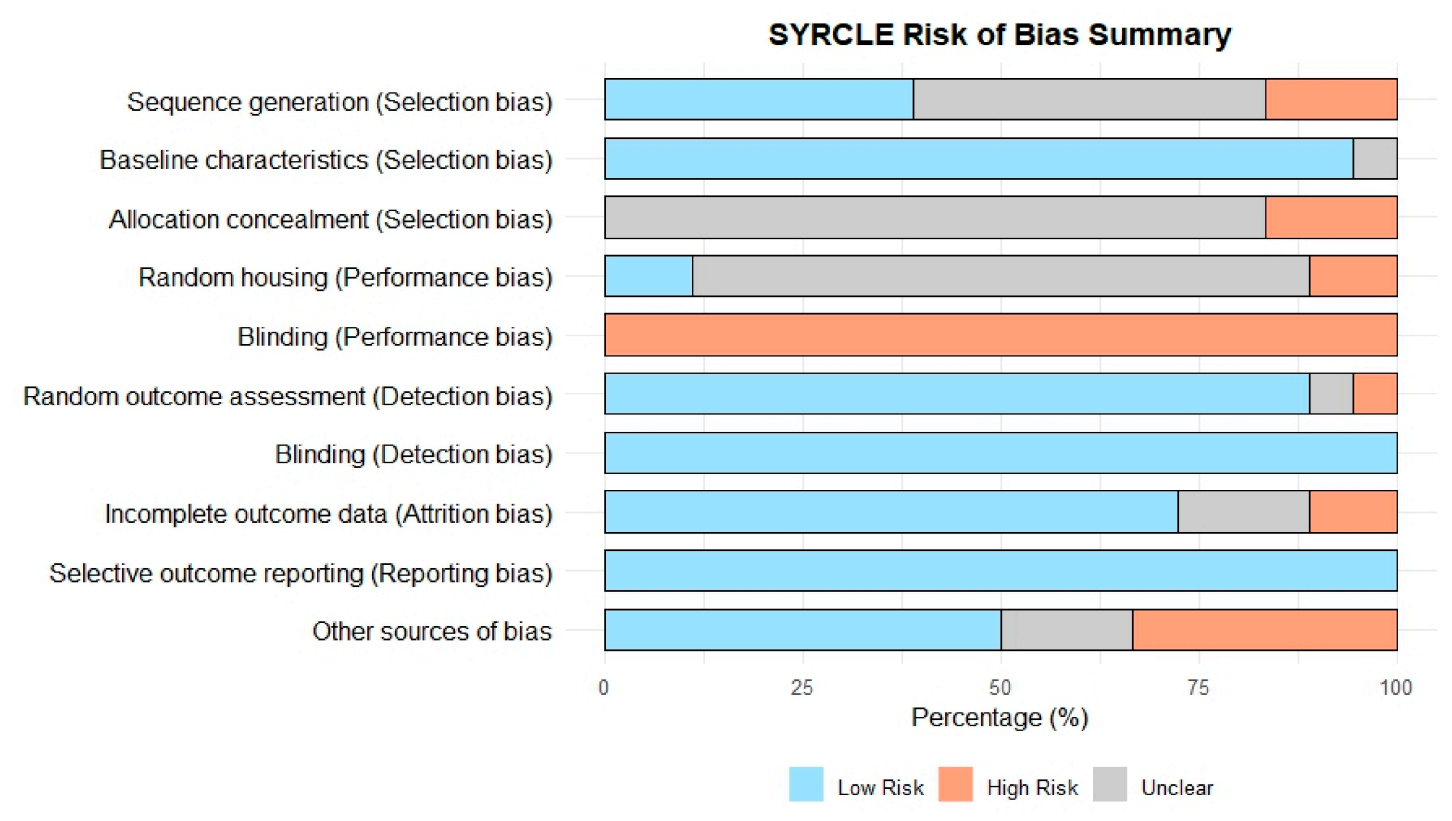
A bar plot summarizing the assessment of risk of bias across the ten SYRCLE domains for the included studies. The horizontal bars indicate the percentage of judgments across studies: light blue represents low risk of bias, orange represents high risk of bias, and grey represents unclear risk of bias.

The risk of bias assessment for the 18 studies, conducted using the SYRCLE’s RoB tool, is presented in Figure 2. For sequence generation (Domain 1), 7 of the 18 studies (39%) demonstrated low bias risk, 8 (44%) showed unclear bias risk, and 3 (17%) exhibited high bias risk. Regarding baseline characteristics (Domain 2), 17 studies (94%) showed low bias risk, while 1 study (6%) showed unclear bias risk. For allocation concealment (Domain 3), 15 studies (83%) exhibited unclear bias risk, and 3 studies (17%) showed high bias risk. In random housing (Domain 4), 14 studies (78%) showed unclear bias risk, while low and high bias risks were each identified in 2 studies (11%). Blinding (Domain 5) was rated as high bias risk in all included studies. For random outcome assessment (Domain 6), 16 studies (89%) demonstrated low bias risk, whereas unclear and high bias risks were each identified in 1 study (5%). All studies showed low bias risk for blinding (Domain 7) and selective outcome reporting (Domain 9). For incomplete outcome data (Domain 8), 13 studies (72%) showed low bias risk, 3 (17%) showed unclear bias risk, and 2 (11%) showed high bias risk. Finally, for other sources of bias (Domain 10), 9 studies (50%) exhibited low bias risk, 3 (17%) exhibited unclear bias risk, and 6 (33%) exhibited high bias risk.

### 3.3. Meta-Analysis and Meta-ANOVA (Subgroup Analysis)

#### 3.3.1. Body Weight

**Figure 3:**
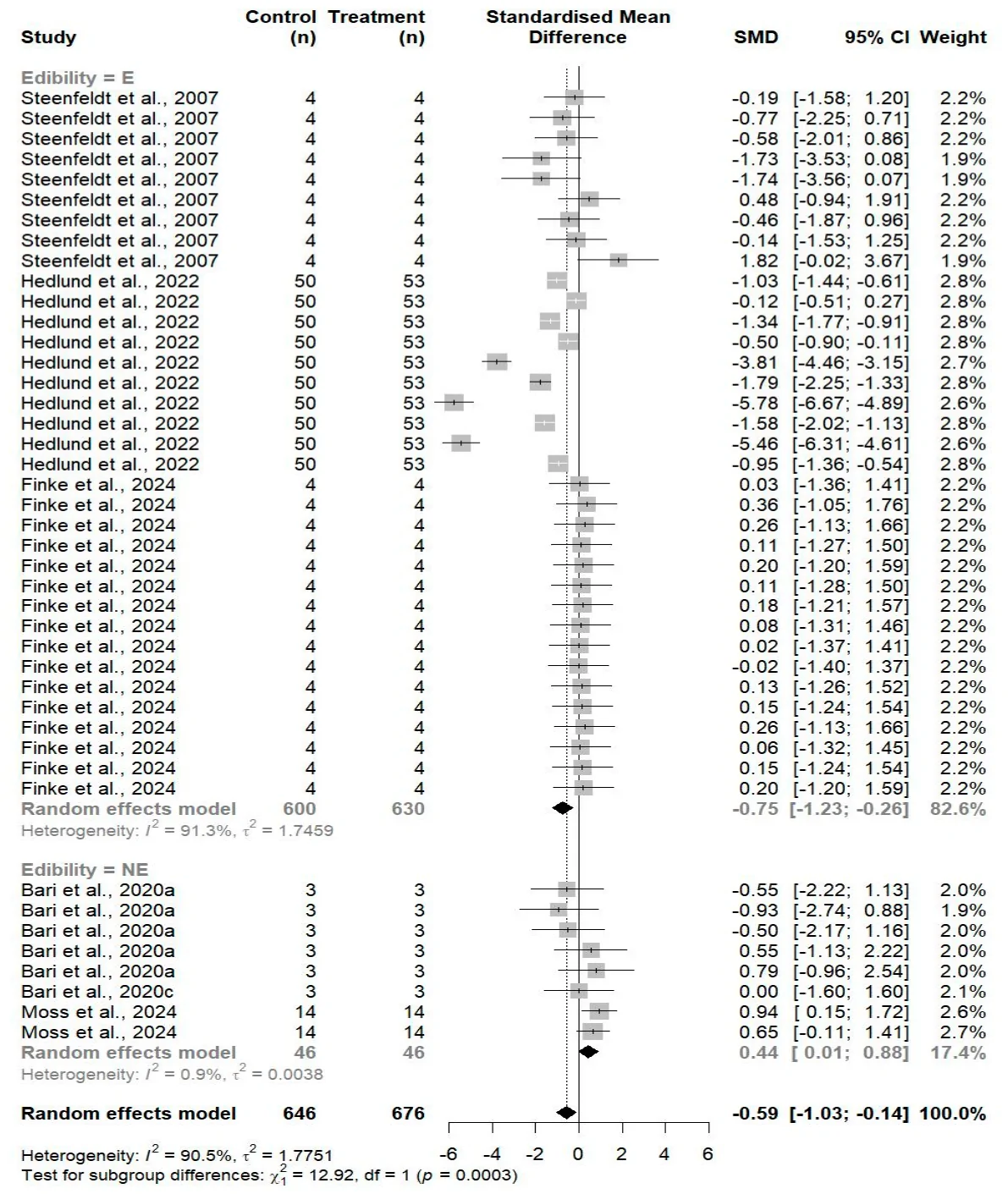
Overall and subgroup effects of pecking materials provision on body weight in laying hens. Grey squares represent individual effect sizes with horizontal lines indicating 95% confidence intervals (the square size reflects the study weighting). Black diamonds represent pooled effect sizes with 95% confidence intervals. The vertical solid line indicates no effect (SMD = 0), and the vertical dashed line indicates the overall pooled estimate [13,16,40,43,45,50].

The effects of pecking materials provision on BW in laying hens are presented in Figure 3. Overall, a decrease in BW was observed with pecking materials provision within a very high level of heterogeneity (SMD = −0.59; 95% CI: [−1.03, −0.14]; *I*^2^ = 90.5%; τ^2^ = 1.7751). Subgroup analysis based on edibility revealed that hens provided edible pecking materials showed a decrease in BW accompanied by very high heterogeneity (SMD = −0.75; 95% CI: [−1.23, −0.26]; *I*^2^ = 91.3%; τ^2^ = 1.7459), whereas hens provided non-edible pecking substances showed an increase in BW with negligible heterogeneity (SMD = 0.44; 95% CI: [0.01, 0.88]; *I*^2^ = 0.9%; τ^2^ = 0.0038).

#### 3.3.2. Egg Production Rate

**Figure 4:**
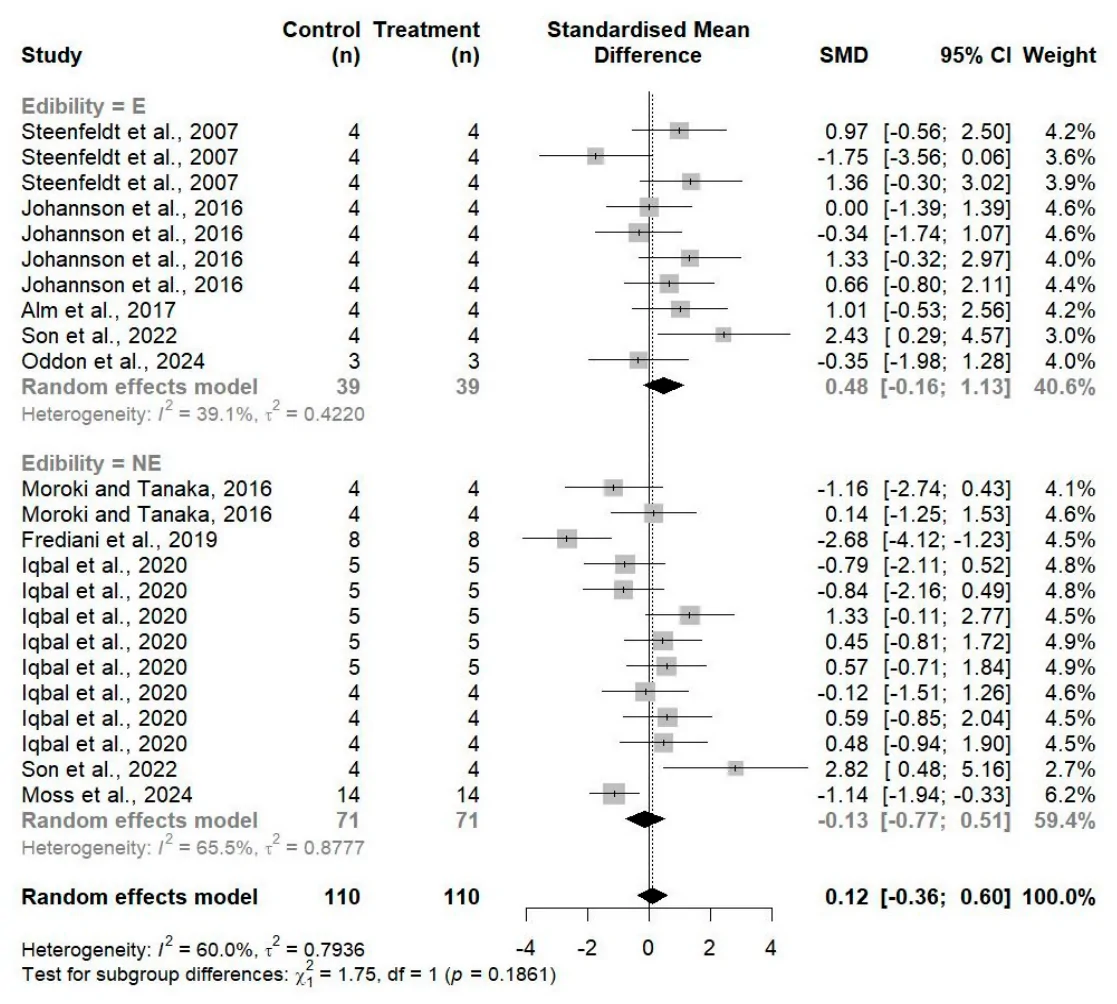
Overall and subgroup effects of pecking materials provision on egg production rate in laying hens. Grey squares represent individual effect sizes with horizontal lines indicating 95% confidence intervals (the square size reflects the study weighting). Black diamonds represent pooled effect sizes with 95% confidence intervals. The vertical solid line indicates no effect (SMD = 0), and the vertical dashed line indicates the overall pooled estimate [4,11,14,40,42,45,47,49].

The effects of pecking materials provision on EPR are presented in Figure 4. The provision of pecking materials had no significant effect on the EPR in laying hens (SMD = 0.12; 95% CI: [−0.36, 0.60]). This result was consistent in subgroup analyses based on edibility, for both edible (SMD = 0.48; 95% CI: [−0.16, 1.13]) and non-edible pecking substances (SMD = −0.13; 95% CI: [−0.77, 0.51]). Overall, heterogeneity was high (*I*^2^ = 60.0%; τ^2^ = 0.7936). In the subgroup analysis, heterogeneity decreased to a moderate level (*I*^2^ = 39.1%; τ^2^ = 0.4220) for edible pecking materials, whereas non-edible pecking materials showed increased heterogeneity (*I*^2^ = 65.5%; τ^2^ = 0.8777).

#### 3.3.3. Egg Mass

**Figure 5:**
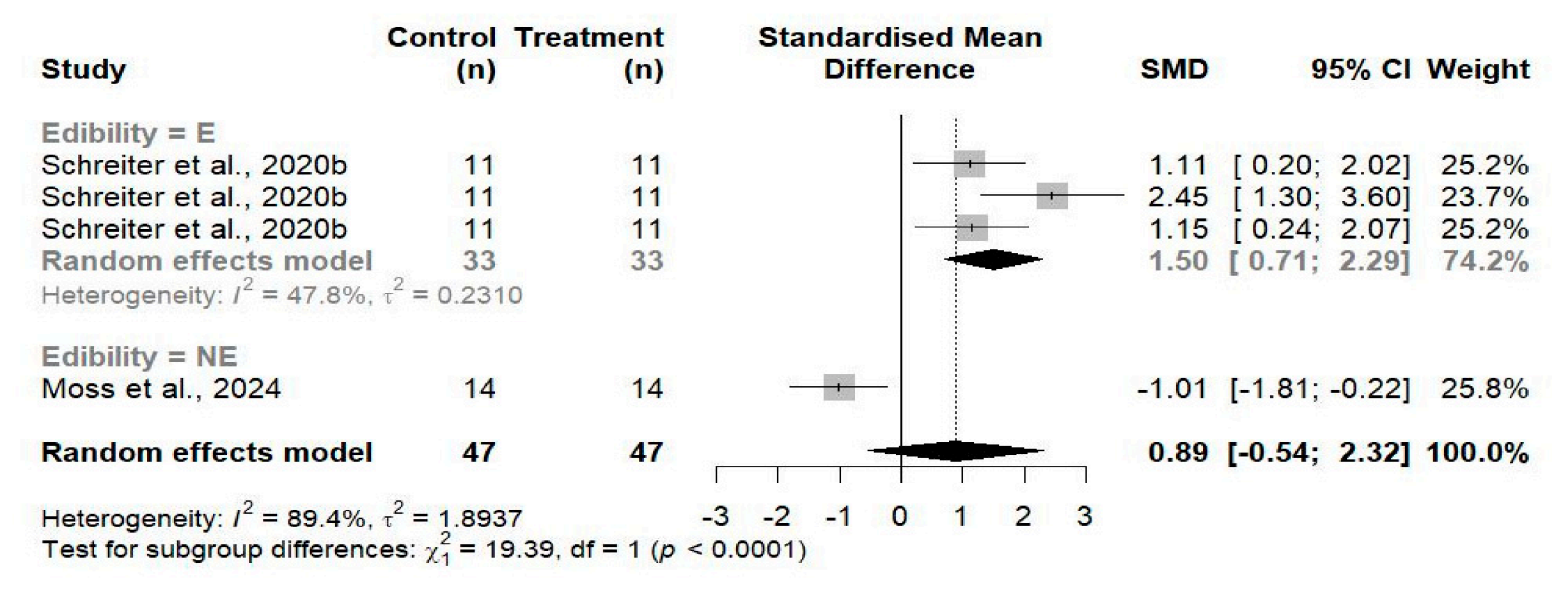
Overall and subgroup effects of pecking materials provision on egg mass in laying hens. Grey squares represent individual effect sizes with horizontal lines indicating 95% confidence intervals (the square size reflects the study weighting). Black diamonds represent pooled effect sizes with 95% confidence intervals. The vertical solid line indicates no effect (SMD = 0), and the vertical dashed line indicates the overall pooled estimate [15,45].

The effects of pecking materials provision on the EM in laying hens are presented in Figure 5. Pecking materials provision had no overall significant effect on EM (SMD = 0.89; 95% CI: [−0.54, 2.32]). However, subgroup analysis revealed a significant increase in EM following the provision of edible pecking materials (SMD = 1.50; 95% CI: [0.71, 2.29]). The heterogeneity (*I*^2^) was reduced from 89.4% (τ^2^ = 1.8937) overall to 47.8% (τ^2^ = 0.2310) when considering only edible pecking materials.

#### 3.3.4. Average Daily Feed Intake

**Figure 6:**
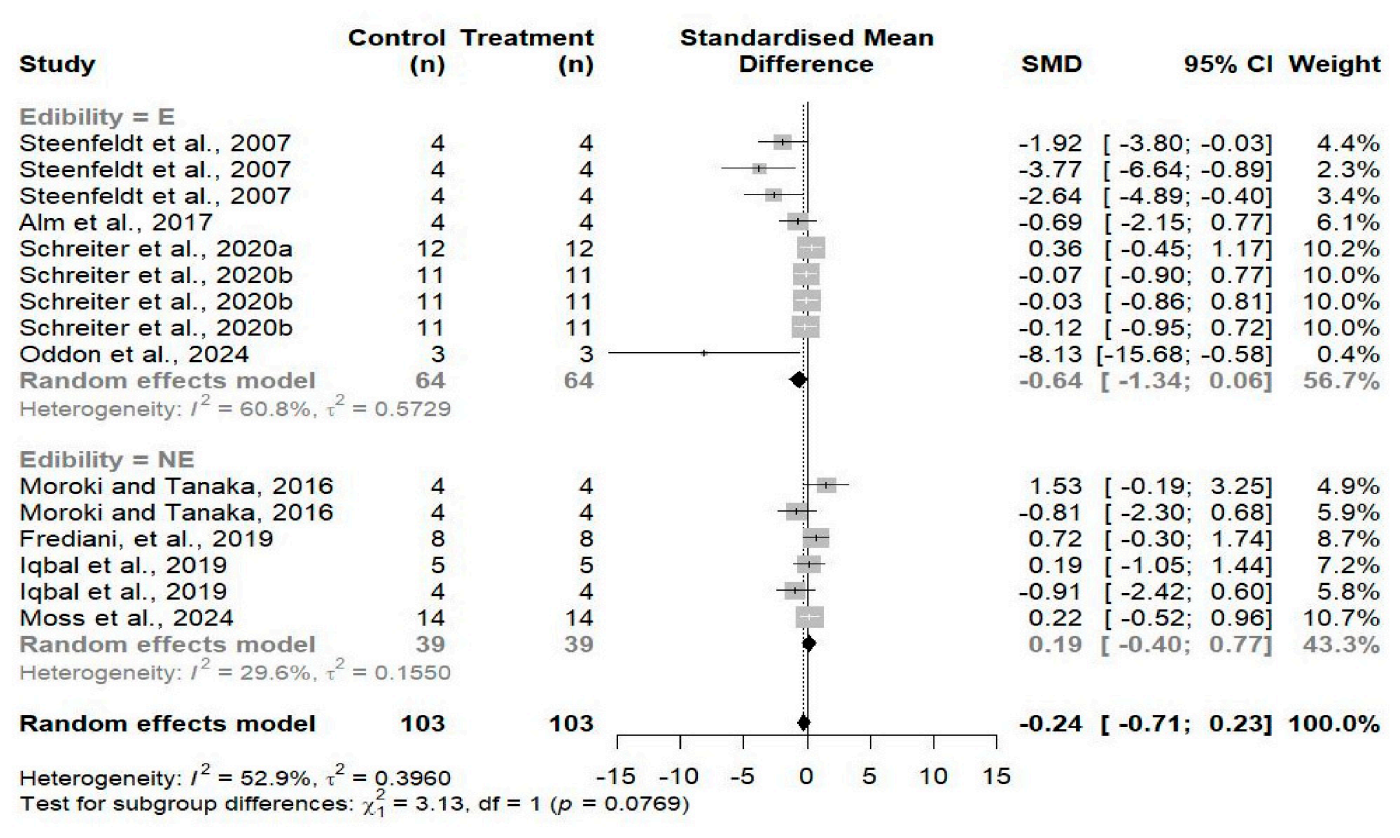
Overall and subgroup effects of pecking materials provision on average daily feed intake in laying hens. Grey squares represent individual effect sizes with horizontal lines indicating 95% confidence intervals (the square size reflects the study weighting). Black diamonds represent pooled effect sizes with 95% confidence intervals. The vertical solid line indicates no effect (SMD = 0), and the vertical dashed line indicates the overall pooled estimate [4,14,15,39,40,41,42,45,48].

The effects of pecking materials provision on ADFI in laying hens are presented in Figure 6. No significant effects were observed in either the overall or subgroup analyses. Overall, heterogeneity was high (*I*^2^ = 52.9%; τ^2^ = 0.3960), which was also observed for edible pecking substances (*I*^2^ = 60.8%; τ^2^ = 0.5729). However, heterogeneity decreased to a moderate level (*I*^2^ = 29.6%; τ^2^ = 0.1550) for non-edible pecking substances.

#### 3.3.5. Feed Conversion Ratio

**Figure 7:**
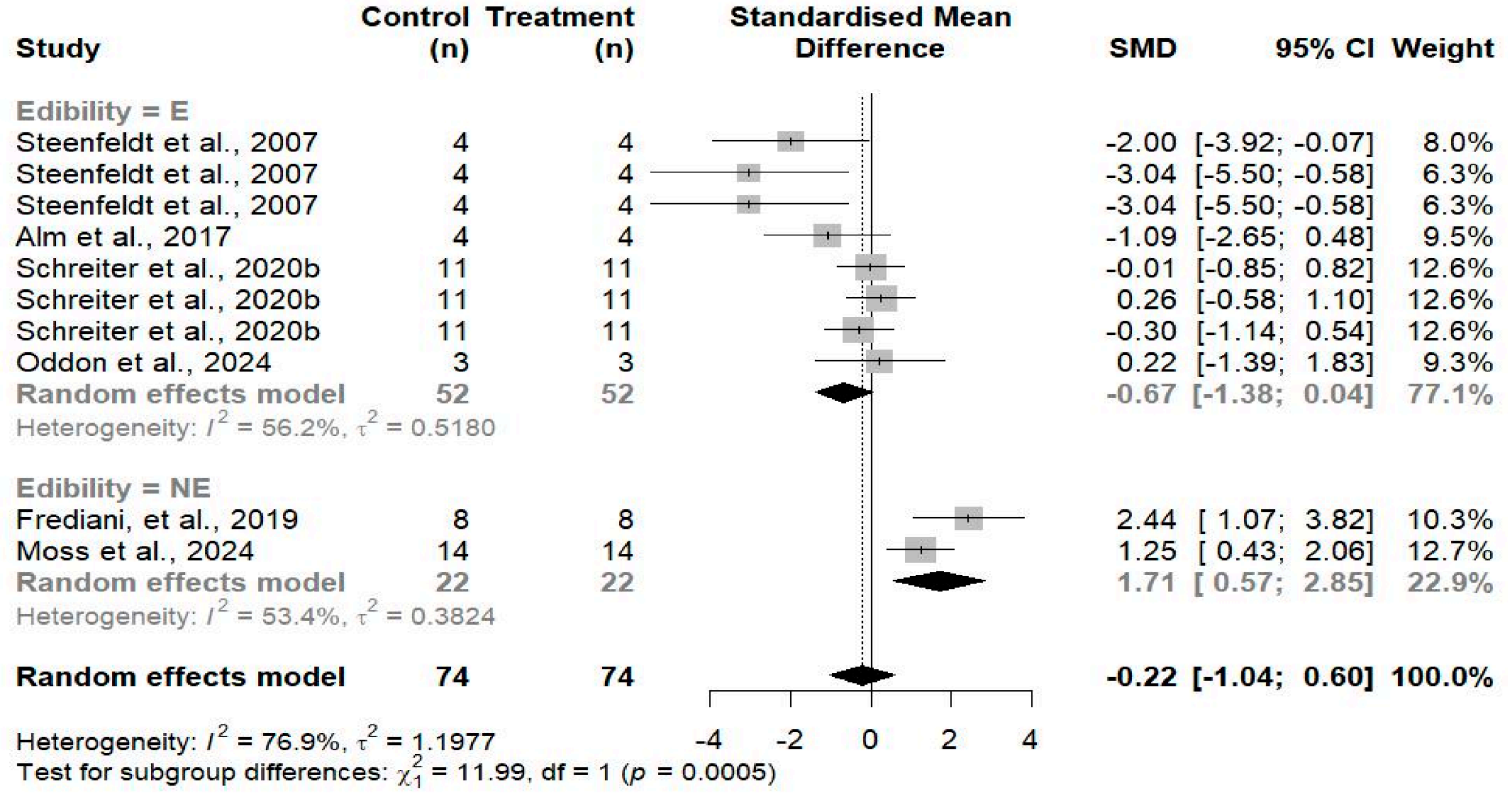
Overall and subgroup effects of pecking materials provision on feed conversion ratio in laying hens. Grey squares represent individual effect sizes with horizontal lines indicating 95% confidence intervals (the square size reflects the study weighting). Black diamonds represent pooled effect sizes with 95% confidence intervals. The vertical solid line indicates no effect (SMD = 0), and the vertical dashed line indicates the overall pooled estimate [14,15,40,41,42,45].

The effects of pecking materials provision on FCR in laying hens are presented in Figure 7. No significant effect on FCR was observed either in the overall analysis (SMD = −0.22; 95% CI: [−1.04, 0.60]) or in the subgroup of edible materials (SMD = −0.67; 95% CI: [−1.38, 0.04]). In contrast, the subgroup of non-edible materials was significantly increased in FCR (SMD = 1.71; 95% CI: [0.57, 2.85]). Overall, heterogeneity was very high (*I*^2^ = 76.9%; τ^2^ = 1.1977); it decreased substantially, ranging from 53.4% to 56.2%, in both subgroup analyses.

#### 3.3.6. Egg Weight

**Figure 8:**
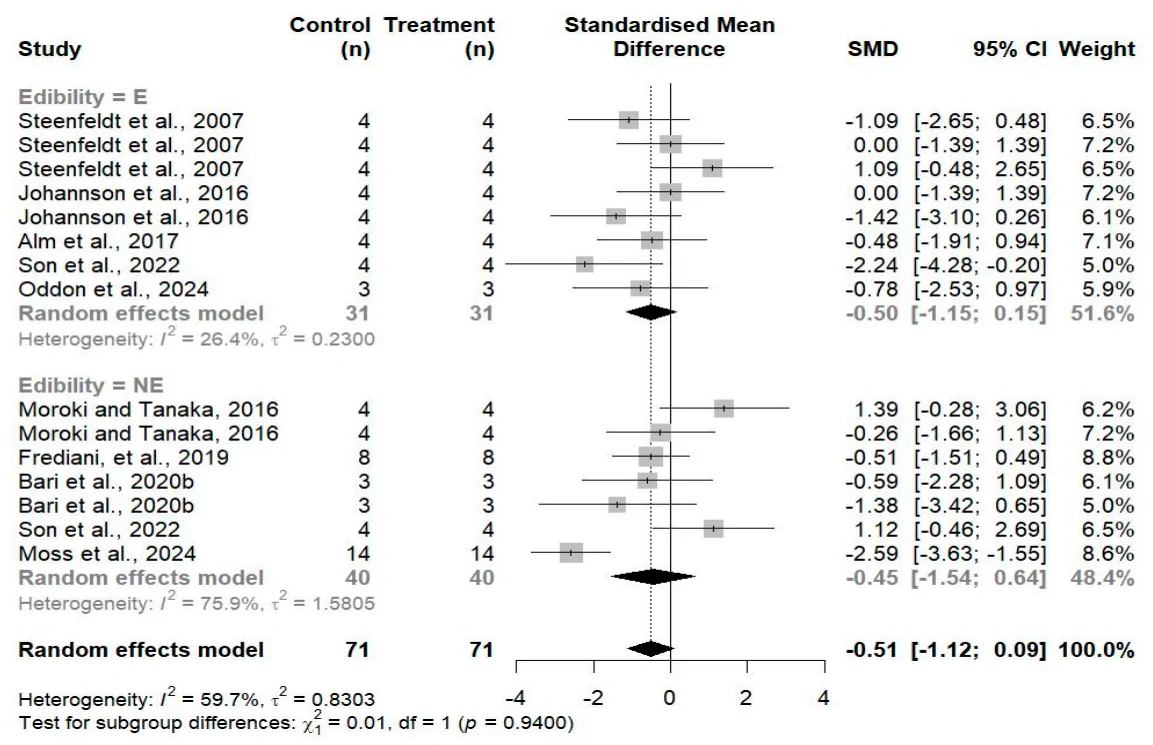
Overall and subgroup effects of pecking materials provision on egg weight in laying hens. Grey squares represent individual effect sizes with horizontal lines indicating 95% confidence intervals (the square size reflects the study weighting). Black diamonds represent pooled effect sizes with 95% confidence intervals. The vertical solid line indicates no effect (SMD = 0), and the vertical dashed line indicates the overall pooled estimate [4,11,14,40,41,42,45,46,47].

The effects of pecking materials provision on EW are presented in Figure 8. The provision of pecking materials had no significant effect on the EW in laying hens (SMD = −0.51; 95% CI: [−1.12, 0.09]). This result was consistent in subgroup analyses based on edibility, for both edible (SMD = −0.50; 95% CI: [−1.15, 0.15]) and non-edible pecking substances (SMD = −0.45; 95% CI: [−1.54, 0.64]). In heterogeneity, both the overall analysis (*I*^2^ = 59.7%; τ^2^ = 0.8303) and the subgroup of non-edible materials (*I*^2^ = 75.9%; τ^2^ = 1.5805) showed high heterogeneity, whereas there was moderate heterogeneity in the subgroup of edible materials (*I*^2^ = 26.4%; τ^2^ = 0.2300).

#### 3.3.7. Mortality Rate

**Figure 9:**
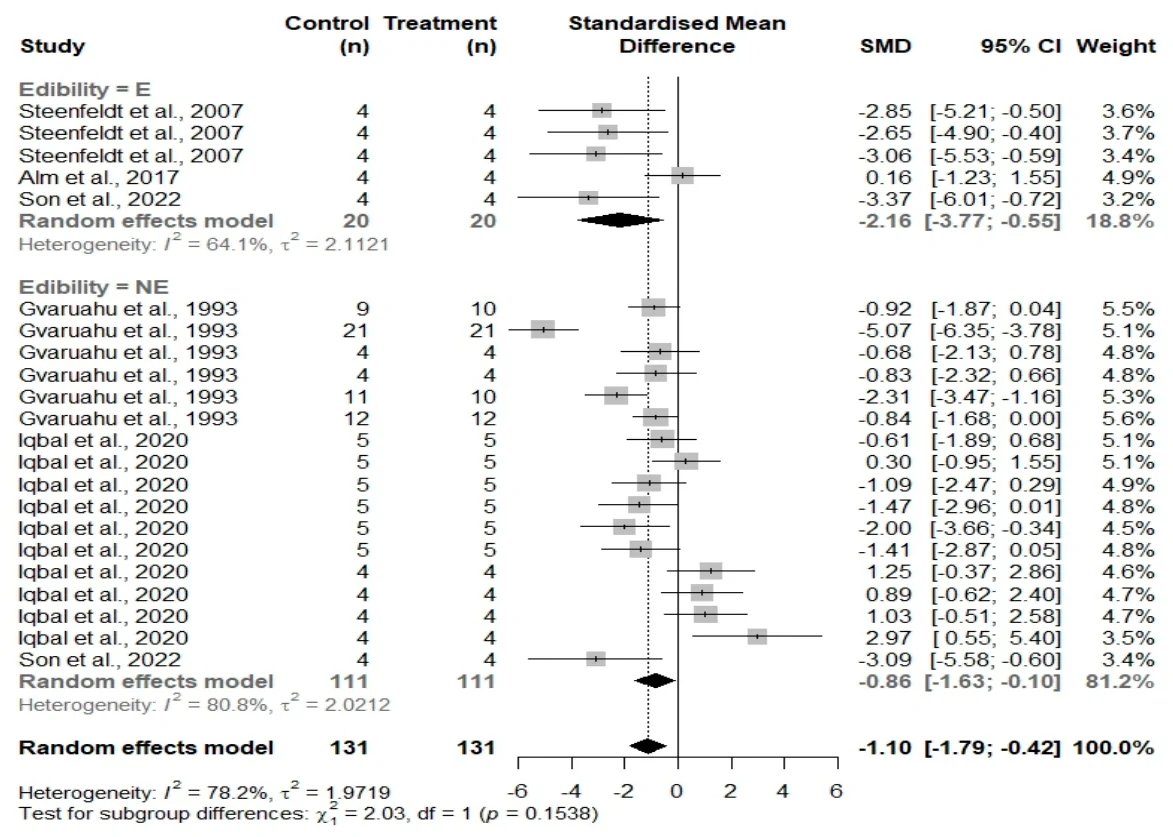
Overall and subgroup effects of pecking materials provision on mortality in laying hens. Grey squares represent individual effect sizes with horizontal lines indicating 95% confidence intervals (the square size reflects the study weighting). Black diamonds represent pooled effect sizes with 95% confidence intervals. The vertical solid line indicates no effect (SMD = 0), and the vertical dashed line indicates the overall pooled estimate [11,40,41,44,49].

The effects of pecking materials provision on MORT are presented in Figure 9. Overall, the provision of pecking materials significantly decreased MORT (SMD = −1.10; 95% CI: [−1.79, −0.42]). This significant decrease was consistent regardless of edibility, being observed for both edible (SMD = −2.16; 95% CI: [−3.77, −0.55]) and non-edible pecking substances (SMD = −0.86; 95% CI: [−1.63, −0.10]). Although the magnitude of the effect was numerically larger for edible materials, the test for subgroup differences indicated no significant difference between the two subgroups (*p* > 0.05). Heterogeneity (*I*^2^) was very high across all analyses, ranging from 64.1% to 80.8% (τ^2^ = 1.9719–2.1121) [11,40,41,44,49].

### 3.4. Assessment of Publication Bias

**Figure 10:**
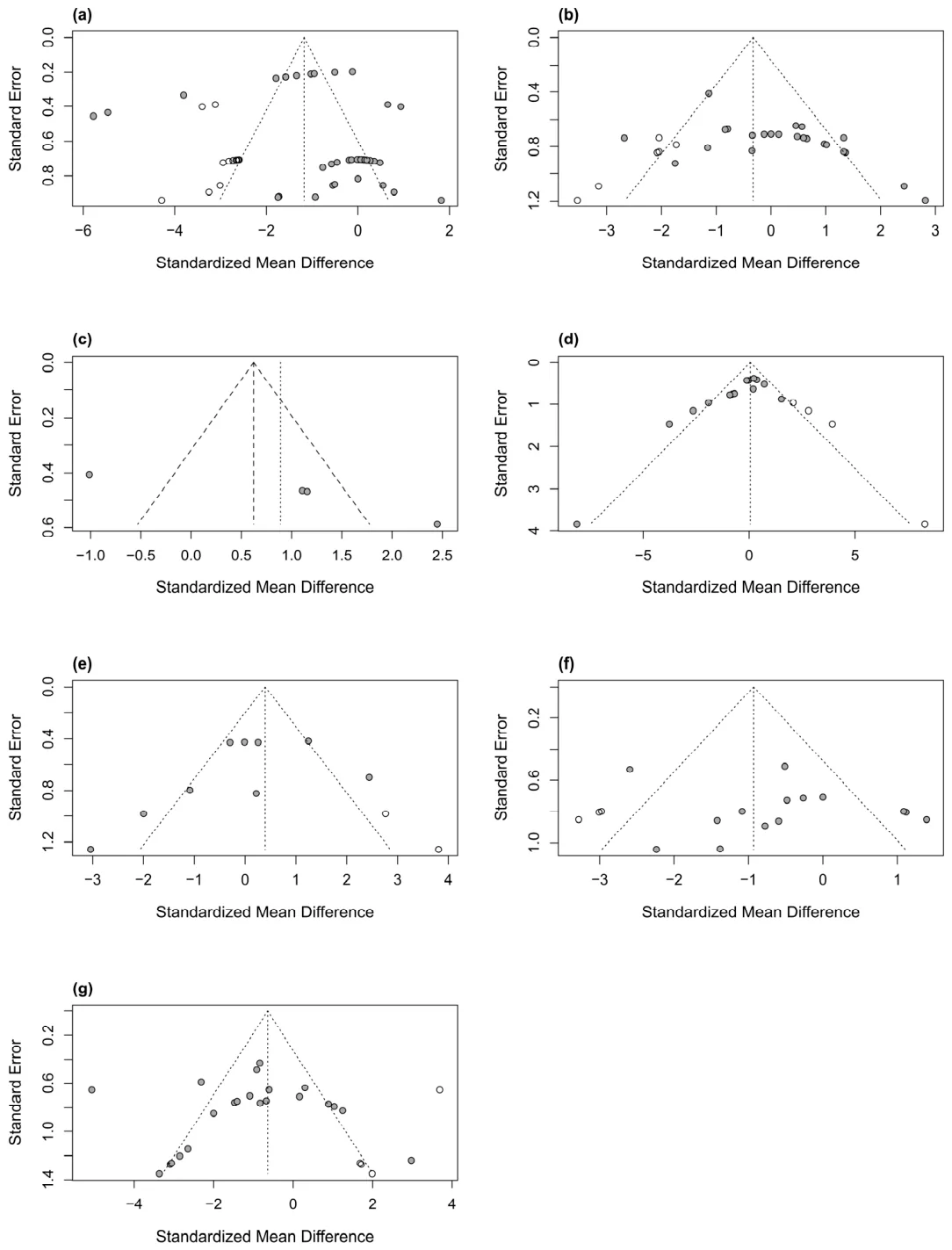
Funnel plots to present publication bias. (**a**): Body weight; (**b**): Egg production rate; (**c**): Egg mass; (**d**): Average daily feed intake; (**e**): Feed conversion ratio; (**f**): Egg weight; (**g**): Mortality. Open circles represent individual studies. The vertical dotted line indicates the pooled effect size, and the diagonal dashed lines represent the 95% pseudo-confidence limits.

The results of publication bias were presented in Figure 10 and Table 2. Asymmetry was visually identified in the funnel plots for EPR and ADFI, with grey data points clustering toward the right or left sides. This was statistically confirmed by Egger’s linear regression test, which indicated significant publication bias for both EPR and ADFI (*p* < 0.05). No significant publication bias was observed for BW and MORT, which showed significant effects of pecking material provision in the meta-analysis, or for FCR and EW, which showed no significant effects (*p* > 0.05).

Publication bias analysis was not performed for EM due to an insufficient number of studies (n = 4), below the minimum threshold (n = 10) generally recommended for Egger’s linear regression test.

The trim-and-fill analysis was performed to estimate the number of potentially missing studies and to obtain adjusted effect sizes for each indicator, as presented in Table 3. For EPR and ADFI, in which publication bias was detected, 6 and 4 studies were imputed, respectively. Following adjustment, the effect sizes remained statistically non-significant for both EPR (SMD = −0.3255; 95% CI: [−0.8244, 0.1734]) and ADFI (SMD = 0.0538; 95% CI: [−0.4988, 0.6064]). Heterogeneity remained high for both indicators after adjustment (*I*^2^ = 68.9% and 65.4%, respectively).

## 4. Discussion

The awareness of poor rearing environments of farm animals and the need for improvement have accelerated research to improve animal welfare levels [4,11,14,51]. In the poultry industry, there are constant efforts to prevent and reduce damage caused by FP, with ethical and social issues surrounding beak trimming, and one of the methods is to provide pecking materials [24]. As can be seen from the literature collected and utilized, approximately 90% of the literature was published after 2015, suggesting that the poultry industry has recently shown a high level of interest in the effects of pecking materials provision. Among these, most studies that systematically reviewed the provision of pecking materials to laying hens as of 2025 only evaluated FP behavior and feather damage (FD), and none analyzed the effect on improving productivity. Therefore, we conducted a meta-analysis to summarize and verify the effects of pecking materials supply on productivity indicators in laying hen housing facilities.

### 4.1. Risk of Bias

Evaluating the methodological validity of individual studies is a critical step in drawing reliable conclusions from a systematic review [26]. In this study, the risk of bias of the 18 included studies was assessed using SYRCLE’s RoB tool [26], which was specifically developed for animal intervention studies.

The assessment showed that approximately 25% of all domain judgments indicated unclear risk of bias and 19.4% indicated high risk of bias. The selection bias and performance bias domains, in particular, showed high proportions of unclear or high-risk judgments. Livestock and animal studies have often been reported to provide insufficient information on group allocation methods, randomization, blinding, and intervention protocols [52], and similar limitations were observed here. In particular, because livestock studies are often conducted on commercial or experimental farms, random allocation and random housing of animals are difficult to implement, which likely contributed to the high proportions of unclear or high-risk judgments in domain 3 (allocation concealment) and domain 4 (random housing). In addition, the present review included studies that provided physical enrichment materials, such as pecking substances, within laying hen housing facilities to compare their effects. As treatment allocation was inevitably distinguishable by visual inspection, all included studies were judged at high risk of bias for domain 5 (blinding).

In contrast, most studies were judged to be at low risk of bias for detection bias, attrition bias, and reporting bias. All evaluated studies reported the full set of analyzed outcomes, and because outcome measures such as mortality and productivity are less susceptible to subjective observer influence, domain 7 (blinding) and domain 9 (selective outcome reporting) were judged to be at low risk of bias across the included studies. Also, most studies either conducted a complete census of productivity outcomes or clearly stated the reasons for excluding individual animals, such as mortality, illness, or regrouping, which likewise contributed to the overall low risk of bias in this category.

Studies with unclear allocation concealment or unclear randomization of housing conditions may not have fully controlled for experimental conditions, such as differences in flock characteristics or unintentional researcher involvement, which may have contributed to the heterogeneity observed among effect sizes. Moreover, due to the limited number of included studies, subgroup or sensitivity analyses stratified by risk-of-bias level could not be performed. Therefore, the potential influence of high-risk studies on the pooled effect sizes cannot be ruled out, and this methodological variability should be considered when interpreting the meta-analysis discussed below.

### 4.2. Effects of Pecking Material Provision and Edibility on Productivity Indicators in Laying Hens

In laying hens, feed intake and body weight are not merely indicators of growth but important parameters reflecting nutrient intake and metabolic status, both of which are closely associated with sexual maturation and, in turn, with subsequent laying performance [53]. Accordingly, adequate feed provision and the maintenance of a body weight consistent with strain-specific standards are considered advantageous from a productivity standpoint. However, our results showed that pecking materials provision had no significant effect on ADFI, whereas BW decreased significantly. Given the high heterogeneity identified in this analysis and the potential contribution of additional nutrients ingested during pecking to BW, subgroup analysis was performed based on the edibility of the materials provided.

Regardless of edibility, ADFI in laying hens showed no significant change with the provision of pecking materials. For edible materials, feed intake decreased as the ingested materials, depending on their nutritional composition, replaced formulated feed [14,40]. However, Alm et al. [41] reported that feed consumption figures were probably overestimated due to feed waste created by hens scratching feed out of the feed hoppers, which ended up in the manure beneath the slatted floor. For non-edible materials, ADFI showed no significant effect in most studies, as no additional material could be ingested [39,42]. In addition to material edibility, differences in rearing conditions may also have influenced ADFI. It has been suggested that the effect of pecking materials provision may not be fully expressed in restricted environments such as cages [42]. Furthermore, Moroki and Tanaka [4] found a significant interaction between device provision and group size on ADFI, with device provision increasing ADFI in singly housed hens but not in pair-housed hens, where the device was more frequently contaminated with feces. These findings suggest that differences in the nutritional composition of edible materials and in rearing conditions contributed to inconsistent directions and magnitudes of ADFI change across individual studies, resulting in a non-significant overall effect size.

Overall, pecking materials provision significantly decreased BW. This reduction, alongside the contrasting effects observed between material types—where edible enrichments significantly reduced BW while non-edible enrichments promoted weight gain—can be elucidated through distinct behavioral and physiological mechanisms. When hens ingest fibrous or moderately nutrient-dense edible materials (such as alfalfa or roughage blocks), the dietary bulk promotes mechanical gut distension and modifies digesta passage rate [43,54]. This physio-chemical satiety satisfies intrinsic foraging and oral exploration motivation without providing excessive caloric density [10]. Consequently, hens exhibit increased exploratory locomotion and foraging activity without surplus caloric deposition, shifting energy partitioning toward maintenance and muscular exercise rather than adipose storage, which ultimately attenuates excessive BW gain [55]. Conversely, interactions with stationary, non-edible objects (such as pumice stones, hanging balls, or plastic devices) redirect abnormal oral behaviors away from conspecifics without altering nutrient intake or digestive physiology. By engaging with these localized environmental cues, hens may reduce widespread, frantic social interactions and aggressive chasing within the flock [44]. This localized engagement dampens overall kinetic energy expenditure and stabilizes metabolic partitioning, allowing energy retention dynamics to favor somatic weight maintenance or slight increases. Moss et al. [45] attributed the BW reduction seen in their pecking materials group to hens exceeding the strain’s standard weight range, interpreting it as a desirable shift away from overweight-related risk. Such findings suggest that BW reduction following pecking materials provision need not always be viewed as an adverse outcome, particularly in overweight flocks. Meanwhile, BW exhibited contrasting patterns depending on the edibility of the materials provided, with edible materials significantly decreasing BW, whereas non-edible materials increased it. However, even within the edible subgroup, BW responses varied across individual studies according to the nutritional properties of the materials provided. Carrots, rich in sugars and soluble non-starch polysaccharides (NSP), and black soldier fly (BSF) larvae, high in fat and protein, provided an energy source and thereby increased BW [14,40], whereas silage, being high in fiber but low in energy density, merely substituted for formulated feed without significantly affecting BW [40]. This suggests that, even among edible materials, the extent and direction of BW change depend on whether the material contributes meaningfully to energy intake. The effect of edible materials on BW is not always determined solely by their contribution to energy supply. Chicks exposed to stress immediately after hatching showed increased utilization of edible materials such as rose hips, bananas, and alfalfa, which was accompanied by higher physical activity and a subsequent decrease in BW [16]. This is interpreted as reflecting stress-induced increases in the motivation to utilize the materials, which in turn elevated activity levels and energy expenditure. For non-edible materials, the underlying mechanism driving BW change was not clearly identified at the level of individual studies. In the hanging CD group of Moss et al. [45], the treatment group showed a numerically higher BW than the control group at the end of the experiment; however, this difference was already present at baseline and therefore cannot be attributed to a physiological effect of the treatment itself. In Bari et al. [13], BW in the treatment group was lower than that in the control group up to 43 weeks of age but reversed after 56 weeks, with a correlation to free-range activity suggested, although the precise underlying mechanism remains unclear. At the level of individual studies, the direction and significance of BW change appear to be determined by multiple factors, including the nutritional composition of the materials, stress-induced motivation to utilize pecking materials, and the activity level of the hens. Nevertheless, the emergence of significant effects in a consistent direction within each subgroup is likely attributable to a statistical property of meta-analysis. As individual studies are pooled, the standard error of the weighted average effect size decreases, narrowing the confidence interval and thereby increasing statistical power, which allows results that were inconsistent at the individual-study level to converge into a significant overall direction [56]. It cannot be ruled out that the subgroup findings of this study likewise reflect this statistical property of meta-analysis. Nevertheless, as observed in the results of Moss et al. [45] and Schreiter et al. [39], BW tended to converge toward the standard range for the strain following the provision of pecking materials, and the possibility that pecking materials provision holds value from the standpoint of body weight management cannot be entirely excluded either.

Despite these results, overall productivity indicators such as EPR, EM, and EW showed no significant effects. Although the overall pooled effect for FCR was non-significant, the non-edible subgroup exhibited a significant increase in FCR (SMD = 1.71). This unfavorable shift in feed efficiency may be linked to increased body weight gain or altered energy expenditure patterns without a corresponding increase in egg mass, warranting careful interpretation. However, some studies have reported significant changes in EPR and EW following the provision of edible [11,40], confirming that the nutritional composition of pecking materials does play a role in productivity and egg quality. From a nutritional and physiological perspective, edible enrichments (e.g., grain- or roughage-based pecking blocks) can provide supplementary metabolizable energy, amino acids, and essential minerals such as calcium and phosphorus [55,57]. Structural fiber intake from these materials stimulates gizzard development, enhances gut motility, and improves nutrient digestibility by optimizing the intestinal microflora [54]. Moreover, the additional supply of bioavailable calcium directly supports the eggshell calcification process in the shell gland, potentially improving eggshell thickness and breaking strength [58], while balanced nutrient utilization can also positively influence internal egg quality parameters such as albumen height and Haugh units [46,59]. In particular, the ingestion of roughage enrichments such as hay or alfalfa bales significantly alters yolk pigmentation and lipid characteristics [40,60]. Forages are naturally abundant in carotenoids (predominantly lutein and zeaxanthin), which are readily absorbed in the avian digestive tract, transported via portomicrons, and deposited directly into the lipid core of the yolk sphere, thereby enhancing yolk color scores without synthetic additives [61]. Furthermore, roughage substrates provide substantial levels of polyunsaturated fatty acids (PUFAs), notably alpha-linolenic acid (ALA, C18:3 n-3) [62]. The voluntary intake of these fibrous materials promotes hepatic elongation and desaturation of dietary ALA into long-chain n-3 fatty acids, culminating in enriched n-3 PUFA profiles (such as EPA and DHA) and a more favorable, reduced n-6/n-3 fatty acid ratio in the yolk lipids [62,63]. Conversely, when high-fiber pecking materials lack sufficient nutritional density, nutrient dilution may occur, potentially mitigating significant improvements in productivity [64]. However, as demonstrated by Johannson et al. [47], providing barley silage in enriched cages successfully redirected injurious pecking without compromising egg production rate or egg weight, even though birds consumed less commercial layer diet. This supports our meta-analytic finding that edible fibrous enrichments can be utilized to satisfy foraging motivation without adversely affecting overall laying performance. BW has also been identified in previous studies as an important factor influencing productivity indicators such as EPR or EW [65,66,67], and it is notable that the BW changes observed in this study did not adversely affect actual laying performance. Differences in BW during early lay have been reported to affect productivity indicators over the course of an extended rearing period [68,69], and further verification is needed to determine whether these non-significant effects would persist over the long term. However, EM was reported in only two studies, yielding four effect sizes, and the non-significant result may be attributable to the limited sample size, warranting cautious interpretation of these findings.

FP among laying hens causes mutual stress and can result in damage to the skin, organs, and reproductive tract, and, in severe cases, mortality [11]. Physiologically, extensive feather loss compromises thermal insulation, dramatically accelerating radiative and convective heat loss from the bare skin [70]. Consequently, affected hens must divert a substantial proportion of dietary metabolizable energy away from egg formation toward thermoregulatory heat production and continuous tissue repair [71]. Furthermore, the persistent physical trauma and social distress induce chronic activation of the hypothalamic–pituitary–adrenal (HPA) axis, leading to sustained elevations in circulating corticosterone and alterations in neuroendocrine signaling, such as central and peripheral serotonin release [72,73]. Elevated systemic corticosterone accelerates skeletal muscle proteolysis and inhibits follicular development and oviductal protein synthesis, while suppressing cellular immunity [74]. This nutrient diversion and metabolic strain ultimately precipitate severe declines in feed conversion efficiency and egg production [69]. Stress hormones such as corticosterone and serotonin released as a result of FP can negatively affect productivity and lead to economic losses on farms [11,24,72]. Accordingly, preventing FP and reducing MORT may be closely associated with improved productivity [75,76]. MORT was effectively reduced in this study simply through the provision of pecking materials, regardless of their edibility. This effect can largely be explained by two mechanisms. One mechanism involves the redirection of pecking motivation from conspecifics toward a novel object. Gvaryahu et al. [44] reported that pecking materials diverted the attention of laying hens in a monotonous cage system, reducing aggressive behavior toward other birds and consequently decreasing MORT. The other involves beak abrasion caused by hard materials, which wear down the beak and thereby reduce the risk of injury to other hens [12,48,49,77]. Iqbal et al. [49] reported that in beak-trimmed hens, the pumice stone group showed improved plumage condition and significantly lower mortality; however, in non-beak-trimmed hens, cumulative mortality was approximately twice as high in the pumice stone group, indicating that beak condition may determine the effectiveness of this mechanism.

Although the marked reduction in heterogeneity within the non-edible BW subgroup (*I*^2^ = 0.9%) suggests that edibility captures part of the variability in productivity responses, heterogeneity remained high across most subgroups and indicators, including MORT, indicating that edibility alone does not fully account for the overall heterogeneity observed in this review. As summarized in Table 1, the included studies also varied considerably in strain, beak-trimming status, housing system, and timing of provision, and several of the findings discussed above suggest that beak condition [48,49] and rearing environment [4,41,50] may have influenced the observed effects. Further studies systematically controlling for these factors are needed to clarify their individual contributions to the heterogeneity observed in this review.

### 4.3. Methodological Considerations and Publication Bias

Publication bias refers to a bias arising from the tendency to preferentially publish significant or favorable results [78], and was assessed in the present study using Egger’s linear regression test and the trim-and-fill method. Although significant bias was detected for EPR and ADFI, its magnitude was not sufficient to alter the overall conclusions of the meta-analysis. In contrast, no evidence of publication bias was observed for BW and MORT, both of which exhibited a significant decrease following the provision of pecking materials.

However, several limitations should be noted, including the absence of grey literature, risk of bias among the included studies, and the limited number of studies. Grey literature, such as theses and reports presenting non-significant or unfavorable results, may not have been fully captured in the search. In addition, the unclear or high risk of bias identified in several domains, combined with the small number of studies available for further stratification, may have partially contributed to the high heterogeneity observed in this review.

Nevertheless, the absence of publication bias evidence for BW and MORT suggests that the significant changes associated with the provision of pecking materials are supported by relatively robust evidence.

## 5. Conclusions

The present meta-analysis evaluated the effects of pecking materials provision on productivity indicators (ADFI, BW, EPR, EM, EW, FCR, and MORT) in laying hens. Pecking materials provision decreased BW and MORT, but had no significant effect on ADFI, EPR, EM, EW, or FCR, supporting the notion that pecking materials can serve as a viable strategy for improving welfare without compromising productivity. Notably, the decrease in MORT was consistent regardless of edibility, whereas BW exhibited contrasting patterns depending on the edibility of the materials provided, suggesting that the inconsistent BW responses reported in previous studies may be partly attributable to the edibility of the materials used. However, the high heterogeneity identified in this study indicates that multiple factors, including material form, nutritional characteristics, and rearing conditions, may have jointly contributed to the observed variability, underscoring the need for future studies that systematically control for these factors.

## Figures and Tables

**Table 1:** Overview of included studies used for the meta-analysis.

References	Strain	Beak Trimming	Housing ^1^	Environmental Enrichments		Productivity Indicator ^3^
				Type	Provision Timing ^2^ (Week of Age)	
Moroki and Tanaka [4]	White Leghorn	-	C	Pecking stones with wooden board	L (65–73)	EPR, ADFI, EW
Son et al. [11]	Hy-Line Brown	No	A	Pecking stones Alfalfa bales	L (40–65)	EPR, EW, MORT
Bari et al. [13]	Hy-Line Brown	Yes	FR	Balls, Bottles, Bricks, Brooms, Brushes, Buckets, Containers, Pet toys, and Plastic pipes	R (0–16)	BW
Oddon et al. [14]	Bionda Piemontese, Bianca di Saluzzo	No	P	Live larvae	L (44–56)	EPR, EW, ADFI, FCR
Schreiter et al. [15]	Lohmann Brown classic, Lohmann Selected Leghorn classic	No	A	Pecking stones, Alfalfa bales	R (1–18); L (19–48)	EM, ADFI, FCR
Hedlund et al. [16]	White Leghorn	-	P	Mealworms, Lettuce, Corn, Rose hips, Alfalfa, Plums, Apples, Pears, Bananas, Cherries and Blackcurrants	R (0–5)	BW
Schreiter et al. [39]	Lohmann Brown classic, Bovans Brown, Lohmann Selected Leghorn classic, Dekalb White	No	A	Pecking stones, Alfalfa bales	R (1–18); L (19–72)	ADFI
Steenfeldt et al. [40]	ISA Brown	No	P	Silage, Barley-pea silage, Carrot	L (20–54)	BW, EPR, EW, ADFI, FCR, MORT
Alm et al. [41]	Dekalb White	No	P	Mussel shells	L (20–72)	EPR, EW, ADFI, FCR, MORT
Frediani et al. [42]	White Lohmann	No	C	White and yellow strings	L (33–42)	EPR, EW, ADFI, FCR
Finke et al. [43]	Lohmann Brown-Lite	No	A	Pecking stones, Alfalfa bales	L (18–78)	BW
Gvaryahu et al. [44]	White Leghorn	-	C	Key rings with colored beads	L (-)	MORT
Moss et al. [45]	Hy-Line Brown	-	C	Hanging CD ^4^	L (24–28)	BW, EPR, EM, ADFI, FCR, EW
Bari et al. [46]	Hy-Line Brown	Yes	FR	Balls, Bottles, Bricks, Brooms, Brushes, Buckets, Containers, Pet toys, and Plastic pipes	R (0–16)	EW
Johannson et al. [47]	White Leghorn	Yes	C	Silage	L (Trial 1: 20–30; Trial 2: 19–28)	EPR, EW
Iqbal et al. [48]	Hy-Line Brown	Both	FR	Pecking stones	L (16–46)	ADFI
Iqbal et al. [49]	Hy-Line Brown	Both	FR	Pecking stones	L (16–46)	EPR, MORT
Bari et al. [50]	Hy-Line Brown	Yes	FR	Balls, Bottles, Bricks, Brooms, Brushes, Buckets, Containers, Pet toys, and Plastic pipes	R (0–16)	BW

^1^ C: cage; P: pen; FR: free-range; A: aviary. ^2^ L: laying period; R: rearing period. ^3^ MORT: mortality; BW: body weight; EPR: egg production rate; EW: egg weight; ADFI: average daily feed intake; FCR: feed conversion ratio; EM: egg mass. ^4^ Compact disk.

**Table 2:** Egger’s linear regression test for publication bias.

Productivity Indicators ^1^	Bias	SE ^2^	*t*-Value	df ^3^	*p*-Value
BW	1.3502	0.9219	1.46	41	0.1506
EPR	3.7907	1.3922	2.72	21	0.0127
EM ^4^	-				
ADFI	−2.4636	0.6648	−3.71	13	0.0026
FCR	−2.8592	1.5512	−1.84	8	0.1025
EW	1.6899	2.0093	0.84	13	0.4155
MORT	−0.5975	1.5938	−0.37	20	0.7117

^1^ BW: body weight; EPR: egg production rate; EM: egg mass; ADFI: average daily feed intake; FCR: feed conversion ratio; EW: egg weight; MORT: mortality. ^2^ SE: standard error of bias estimate. ^3^ df: degrees of freedom. ^4^ Egger’s linear regression test was not performed due to an insufficient number of studies (n < 10).

**Table 3:** The trimmed effect sizes of pecking materials provision on productivity indicators in laying hens.

Productivity Indicators ^1^	df ^2^	Random Effects Model ^3^		Heterogeneity		
		Effect Size	*p*-Value	Q	*I* ^2^	τ
BW	57	−1.1744	<0.0001	574.32	90.1	1.4011
EPR	28	−0.3255	0.2010	90.02	68.9	1.1183
EM ^4^	-					
ADFI	18	0.0538	0.8487	52.08	65.4	0.8977
FCR	12	0.3947	0.3679	61.23	80.4	1.3369
EW	17	−0.9312	0.0057	58.71	71.0	1.1881
MORT	25	−0.6396	0.0995	157.08	84.1	1.7652

^1^ BW: body weight; EPR: egg production rate; EM: egg mass; ADFI: average daily feed intake; FCR: feed conversion ratio; EW: egg weight; MORT: mortality. ^2^ df: degrees of freedom. ^3^ The trim and fill was performed only for indicators with significant publication bias (Egger’s linear regression test, *p* < 0.05). ^4^ Trim and fill analysis was not performed due to an insufficient number of studies (n < 10).

## Data Availability

The datasets presented in this study are available on request from the corresponding author upon reasonable request.

## Abbreviations

FP	Feather pecking
SFP	Severe feather pecking
VP	Vent pecking
EE	Environmental enrichment
BW	Body weight
EPR	Egg production rate
EM	Egg mass
ADFI	Average daily feed intake
EW	Egg weight
FCR	Feed conversion ratio
MORT	Mortality rate
PRISMA	Preferred reporting items for systematic reviews and meta-analyses
SD	Standard deviation
SEM	Standard error of mean
SYRCLE	Systematic review centre for laboratory animal experimentation
SMD	Standardized mean differences
CI	Confidence intervals
REM	Random effects model
NSP	Non-starch polysaccharides
BSF	Black soldier fly
